# Review on Local Buckling of Hollow Box FRP Profiles in Civil Structural Applications

**DOI:** 10.3390/polym13234159

**Published:** 2021-11-28

**Authors:** Mohammad Alhawamdeh, Omar Alajarmeh, Thiru Aravinthan, Tristan Shelley, Peter Schubel, Ali Mohammed, Xuesen Zeng

**Affiliations:** 1 Centre for Future Materials, University of Southern Queensland, Toowoomba, QLD 4350, Australia; Omar.Alajarmeh@usq.edu.au (O.A.); Thiru.Aravinthan@usq.edu.au (T.A.); tristan.shelley@usq.edu.au (T.S.); Peter.Schubel@usq.edu.au (P.S.); Xuesen.Zeng@usq.edu.au (X.Z.); 2 Wagners Composite Fibre Technologies, Wellcamp, QLD 4350, Australia; ali.mohammed@wagner.com.au

**Keywords:** pultruded FRP profiles, local buckling, wall slenderness, cross-sectional aspect ratio, corner geometry, layup properties

## Abstract

Hollow box pultruded fibre-reinforced polymers (PFRP) profiles are increasingly used as structural elements in many structural applications due to their cost-effective manufacturing process, excellent mechanical properties-to-weight ratios, and superior corrosion resistance. Despite the extensive usage of PFRP profiles, there is still a lack of knowledge in the design for manufacturing against local buckling on the structural level. In this review, the local buckling of open-section (I, C, Z, L, T shapes) and closed-section (box) FRP structural shapes was systematically compared. The local buckling is influenced by the unique stresses distribution of each section of the profile shapes. This article reviews the related design parameters to identify the research gaps in order to expand the current design standards and manuals of hollow box PFRP profiles and to broaden their applications in civil structures. Unlike open-section profiles, it was found that local buckling can be avoided for box profiles if the geometric parameters are optimised. The identified research gaps include the effect of the corner (flange-web junction) radius on the local buckling of hollow box PFRP profiles and the interactions between the layup properties, the flange-web slenderness, and the corner geometry (inner and outer corner radii). More research is still needed to address the critical design parameters of layup and geometry controlling the local buckling of pulwound box FRP profiles and quantify their relative contribution and interactions. Considering these interactions can facilitate economic structural designs and guidelines for these profiles, eliminate any conservative assumptions, and update the current design charts and standards.

## 1. Introduction

### 1.1. Background

Pultruded fibre-reinforced polymer (PFRP) profiles have flourished in the last few decades and have become a reliable construction element, especially after the research and development efforts that made pultrusion a more robust and economic manufacturing process [1,2]. These profiles developed from being strengthening and rehabilitating elements to being essential structural members because of their excellent mechanical properties, light weight, and superior corrosion resistance [3,4]. They are currently used as beams [5], decks and panels [6,7,8,9], and trusses [10,11,12] in buildings and bridges, frames in marine structures [13,14,15], lighting poles and cross-arms in infrastructure [16,17], pipes in the oil industry [18,19], spar caps for wind turbines and cable trays and grating walkways in solar structures in the energy sector [20,21], reinforcements for concrete [22,23], piles foundations [24,25], and sleepers in railways [26,27,28].

The introduction of pulwinding technology was one of the most prominent developments in pultrusion. In this process, off-axis wound fibres replace continuous filament mats to be pulled along with the axial fibre rovings, which enables the laminate to reach a higher value of fibre volume fraction with high-quality control and low defects (resin-rich zones) content. The wound fibres improve the transverse properties and delamination resistance and enhance the post-processing endurance, such as jointing and bolting [10,29].

### 1.2. Research Significance

The market share of FRP profiles has increased rapidly in the last decade to reach USD 15.3 billion, which is 6.4% of the construction market [30]. Nevertheless, the current design standards and manuals are still basic and contain only conservative formulas for the design against local buckling with no considerations for the interactions between the design parameters [31]. This lack of knowledge discourages design engineers and contractors from heavily relying on these profiles in infrastructure applications due to uncertainty and overdesign. In addition, the structural design of FRP composites requires more specifications compared to isotropic materials since the layup and geometric parameters have to be assigned for composites while only the dimensions are to be determined for isotropic material [32,33]. Local buckling is a major failure mode controlling the behaviour of PFRP profiles because of their anisotropic and slender nature [34,35]. It can occur before the element reaches its ultimate strength [36,37,38]. The use of box PFRP profiles is still modest compared to the conventional construction materials due to the lack of local buckling design guidelines and manuals accounting for all the design parameters and their interactions [39]. This limitation presents an obstacle in designing these profiles and utilising their potentials.

This article presents a literature review on the local buckling design parameters controlling the structural behaviour of box PFRP profiles. First, the local buckling design of open-section (I, C, Z, L, T shapes) and closed-section (box) FRP structural shapes was reviewed and compared. Second, the critical design parameters were reviewed along with the available literature on each structural shape. Finally, each design parameter was discussed in terms of the interactions with the other parameters (the effect of one parameter on the influence of the other parameter). The article outlines the current state of knowledge and the further investigations to be conducted; thus, it provides a useful reference to design engineers and researchers. Although most of the parameters were studied on the open-section profiles, there is still a need to perform a comprehensive study to obtain the parametric contribution and interaction for box shape pulwound profiles due to their unique stresses’ distribution. Considering these interactions will facilitate more economic and efficient structural designs and guidelines and will result in reliable design charts and recommendations on the design for manufacturing parameters for direct use. Consequently, it will broaden the use of PFRP in civil structural applications.

## 2. Local Buckling in Composites

Pultruded FRP profiles are prone to local buckling failure, well below their ultimate load capacity, due to their anisotropic elasticity and application-driven slenderness [24,40]. Unlike other failure modes, which depend on the material strength, local buckling depends on the stiffness, geometry, and boundary and loading conditions of the element and can occur before reaching the strength limit [37,41,42]. Contrary to ductile and isotropic metals, the local buckling behaviour of FRP composites is different as it is usually accompanied by a growth of cracks and delamination [43,44]. In this literature review, only the design for manufacturing parameters related to the stiffness and geometry of the box FRP profiles is discussed. The other parameters affecting the local buckling of these profiles, such as the boundary condition and geometric imperfection, are out of this review’s scope.

**Figure 1:**
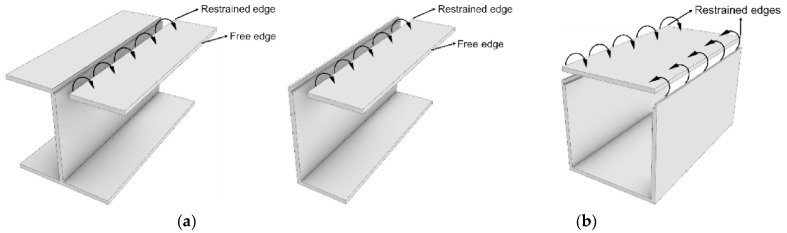
FRP composite profiles with (**a**) open-section and (**b**) closed-section (box) shapes (modified from [57]).

**Figure 2:**
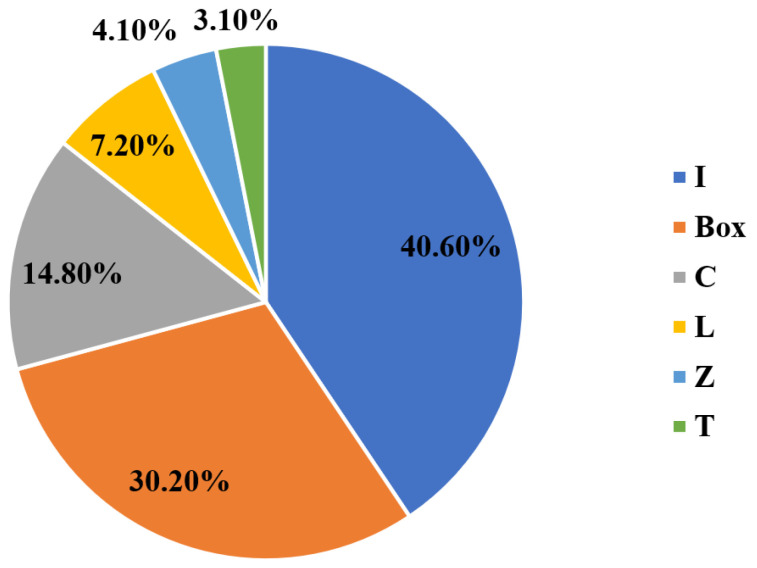
The percentage share of each cross-sectional shape in civil structural applications along with the studies (experimental and numerical) characterising its local buckling behaviour (I-shape: [17,52,53,57,58,59,60,61,62,63,64,65,66,67,68,69,70,71,72,73,74,75,76,77,78,79,80,81,82,83,84,85,86,87,88,89,90,91,92], Box-shape: [17,29,53,54,55,56,58,63,72,75,76,82,85,93,94,95,96,97,98,99,100,101,102,103,104,105,106,107,108], C-shape: [63,75,78,82,85,86,87,97,109,110,111,112,113,114,115], L-shape: [17,63,75,78,85,86,87], Z-shape: [78,85,86,87], and T-shape: [78,85,87]).

**Figure 3:**
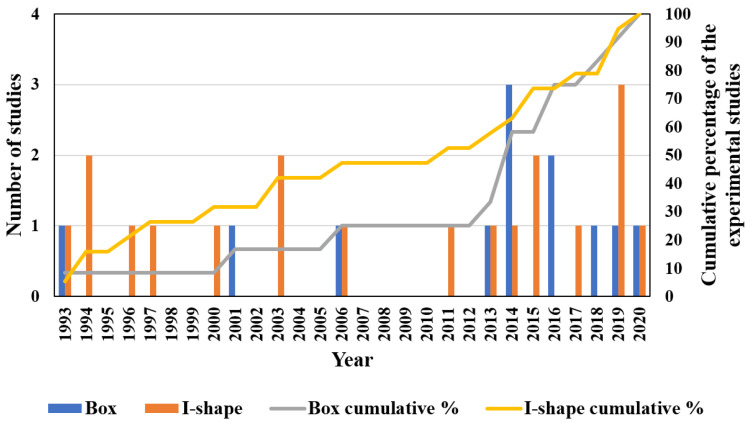
The number of experimental studies of local buckling undertaken on I-shape versus box shape for civil structural applications (Box-shape: [17,29,53,55,56,72,95,96,98,100,101,104] and I-shape: [17,53,61,62,64,66,67,68,69,70,71,72,77,79,81,89,90,91,92]).

The cross-sectional shape of the PFRP profiles controls their structural performance and their dominant failure mode [45,46,47]. Regarding local buckling behaviour, PFRP profiles are categorised into two groups of open-section and closed-section (box) shapes depending on the restraint provided for the flange, as shown in Figure 1. Figure 2 shows the percentage share of each cross-sectional shape in civil structural applications along with the studies characterising its local buckling behaviour. The circular tube shape was not considered here since local buckling is not critical in tubular PFRP profiles used in civil structural applications due to their relatively low slenderness ratio and uniformly distributed stresses [48,49,50,51]. The I-shape is most common in FRP profiles since it was inherited from the steel industry [52,53]. Nevertheless, box profiles are receiving more attention because of their higher structural stability and torsional stiffness with all walls being restrained [54]. Despite that, the majority of the local buckling studies were conducted on I-shape profiles, as shown in Figure 3, which compares the number of experimental studies undertaken on I-shape versus box shape in civil structural applications. The I-shape geometry was studied over three times more frequently than the box shape up to 2014. With the introduction of pulwinding technology for commercial production, the number of studies on box profiles was multiplied in 2014. Only three experimental studies on local buckling of pulwound FRP profiles were undertaken in 2014 [55], 2016 [56], and 2019 [29].

**Figure 4:**
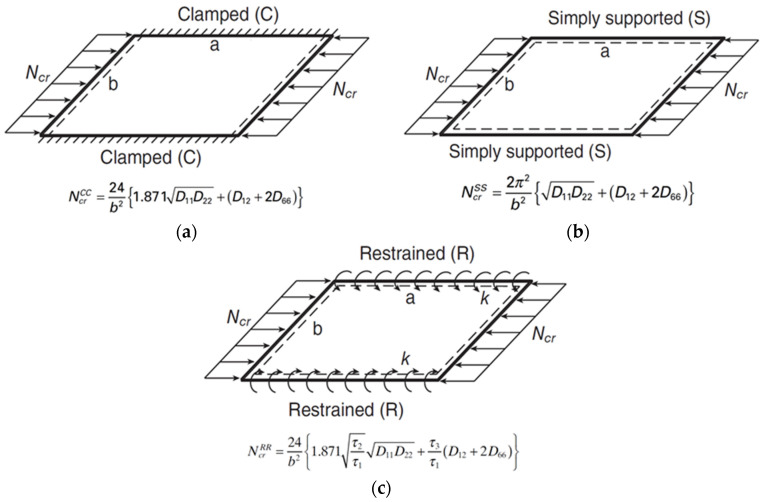
FRP plates of box profile with various unloaded edge conditions: (**a**) clamped, (**b**) simply supported, and (**c**) elastic restrain (modified from [121]).

**Figure 5:**
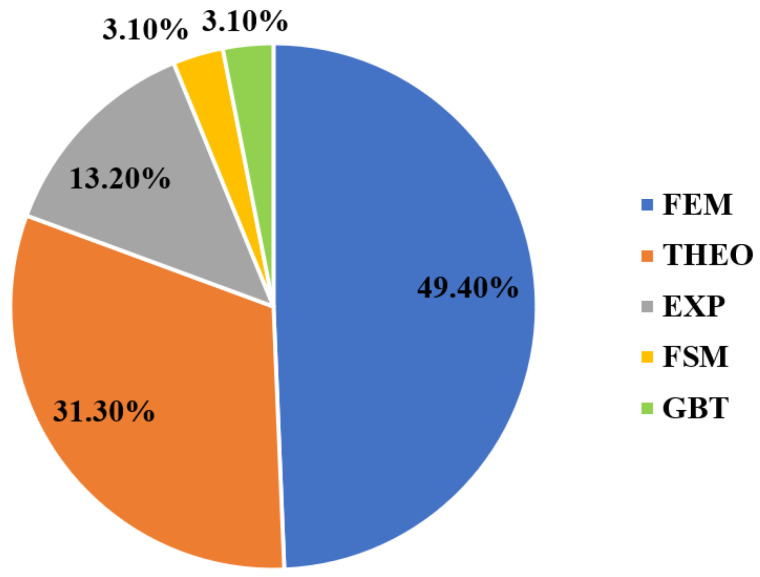
The percentage of each research methodology used to study local buckling and its parameters (FEM: finite element method [29,54,55,57,58,66,68,69,71,73,81,88,91,93,94,95,99,106,108,109,110,111,112,113,114,115,131,132,133,134,135,136,137,138,139,140,141,142,143,144,145,146,147,148,149,150,151,152,153,154,155,156,157,158,159,160], THEO: theoretical approaches [53,54,57,59,60,62,63,64,65,66,67,74,75,76,77,78,80,82,83,84,85,86,87,96,97,105,107,109,135,152,161,162,163,164,165,166,167], EXP: experimental investigations [17,52,56,61,70,72,79,89,90,92,98,100,101,103,104], FSM: finite strip method [63,64,168,169], and GBT: generalised beam theory [65,68,69,80,88]).

Local buckling can be defined as a structural instability problem where the cross-sectional elements (e.g., flange or web) in a compressive loaded member will undergo an out-of-plane deformation and a stiffness reduction, which may lead to structural collapse [116,117,118]. It is a dominant failure mode for short-length FRP profiles and its capacity depends on the elastic properties of the laminate, the geometry, and the supporting and loading conditions of the cross-sectional elements [119,120,121]. Theoretically, local buckling of FRP profiles is analysed by considering each wall (e.g., flange or web) individually as an orthotropic plate and modelling the restraint of the flange-web junctions. Rayleigh–Ritz method is used to approximate the eigenvalue solution of the stability problem depending on the boundary and continuity conditions [85,122]. The theoretical approaches to simulate this restraint (boundary condition) are varying between three assumptions considering the flange-web junction to be clamped, simply supported, or elastically restrained, as shown in Figure 4 for box FRP profile. These three cases represent the upper, lower, and intermediate bounds of the buckling capacity ($N_{cr}$), respectively [123]. The explicit closed-form solutions for these cases are also presented in the same figure, where $D_{11}$, $D_{22}$, $D_{12}$, and $D_{66}$ are the flexural rigidities (the equivalents of EI per unit width) of the orthotropic plate and the coefficients $\tau_{1}$, $\tau_{2}$, and $\tau_{3}$ are functions of the rotational restraint (k) of the flange-web junction. It is worth mentioning that such closed-form equations are based on the classical laminated plate theory (CLPT) which does not count for shear deformations, and they consider only the geometry and layup of the plate [47,124]. They do not account for the flange-web junction (corner) geometry and cannot answer for the interactions with other failure modes. Thus, considering the local buckling of PFRP profiles as a plate instability problem results in inaccurate predictions due to the omission of stresses distribution from the adjacent walls. It is always preferable to consider the whole cross-sectional geometry when analysing buckling problems, and the finite element method (FEM), finite strip method (FSM), and generalised beam theory (GBT) are usually used for this purpose [125,126,127]. Nevertheless, the FEM surpasses the other numerical approaches due to its flexible and accurate simulation of geometry (e.g., tapering or thickening the corner radius). This is evident from the reviewed literature as shown in Figure 5, which shows the percentage of each research methodology used to study local buckling and its parameters. FEM is the best candidate to study the design parameters and perform parametric studies because of its flexibility in handling complex geometries, different loading and boundary conditions, and combined failure problems [128,129,130].

**Figure 6:**
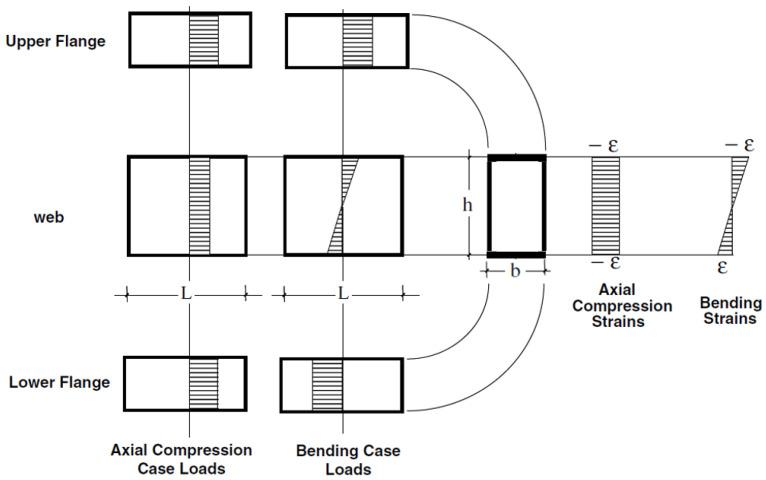
Distribution of strain and load per unit width in the flanges and the web of a box section subjected to axial compression or bending (modified from [86]).

The local buckling behaviour of PFRP profiles varies depending on the loading condition as shown in Figure 6, which depicts the distribution of stress and strain in the hollow box profile subjected to compression versus bending. In profiles subjected to compression, all the walls buckle with a smaller buckle half-wavelength. Whereas in bending, only the walls under compressive stresses will buckle with a larger buckle half-wavelength [45,60,170]. Thus, local buckling is more critical in compression members than in flexural members due to the lower restraint provided by adjacent walls in compression members [24,171,172]. Consequently, investigating and optimising the local buckling behaviour should be undertaken under both loading conditions in which compression provides the upper limit case and bending provides the lower limit case.

**Figure 7:**
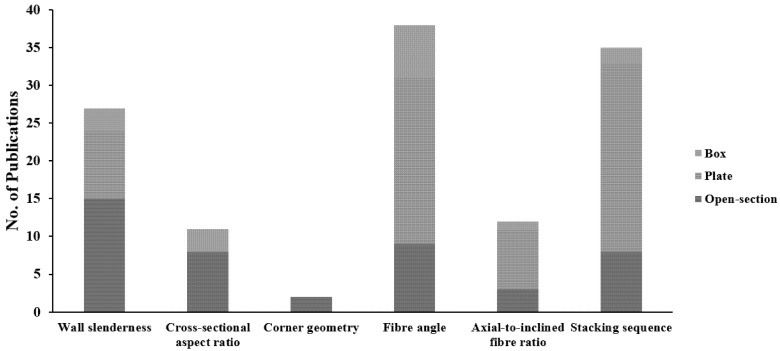
The number of publications on the manufacturing design parameters of local buckling for different FRP composite geometries (Wall slenderness: Open-section [17,66,67,72,75,76,77,81,83,84,87,90,108,109,111], Plate [133,137,143,144,145,146,152,153,169], and Box [54,76,94], Cross-sectional aspect ratio: Open-section [63,64,65,68,69,74,86,111] and Box [63,106,108], Corner geometry: Open-section [61,89], Fibre angle: Open-section [53,58,74,78,80,87,97,113,114], Plate [131,132,133,134,136,139,141,142,143,145,146,147,148,149,151,153,155,156,163,165,166,168], and Box [29,53,54,58,94,95,97], Axial-to-inclined fibre ratio: Open-section [80,87,97], Plate [136,137,141,147,149,153,155,168], and Box [94], and Stacking sequence: Open-section [80,87,97,109,111,112,114,140], Plate [131,135,136,137,138,141,142,144,145,146,147,148,149,151,153,155,156,159,160,162,163,164,165,166,167], and Box [54,97]).

The critical manufacturing design parameters controlling the local buckling behaviour of FRP composites can be categorised into two groups of geometric (wall slenderness, cross-sectional aspect ratio, and corner geometry) and layup parameters (axial-to-inclined fibre ratio, inclined fibre angle, and stacking sequence) [124,173,174,175,176]. After reviewing the available literature, it appears that these design parameters were not comprehensively studied for closed-section geometry (box profiles) when compared to other geometries, as shown in Figure 7, evident by the minimum number of publications for each manufacturing design parameter. Moreover, no study was found to investigate the corner radius effect on the local buckling capacity and failure mode of box profiles. Most of the publications on the layup parameters were undertaken for laminated plate geometry, not structural-level shapes. The effect of the layup parameters on the corners, which represent critical failure zones, was not considered in such studies. Table 1 summarises the local buckling design formulas of compression box and I-shape members in current standards and guides [177,178,179,180]. The effect of the cross-sectional aspect ratio is neglected in [177], which relies on the maximum slenderness ratio only. Reference [179] does not consider the effect of the rotational restraint between the flange and web. All the design standards neglect the corner radius in their local buckling design formulas. These design parameters should be studied in combination to obtain their contribution and interactions, allowing for a better understanding of the structural performance of box profile geometry and its unique stresses distribution. Consequently, this will enhance the current standards and make them more accurate by considering the corner geometry and its interactions with the other design parameters in the design formulas of these standards.

The boundary and interaction between local and global buckling modes were extensively investigated for both open-section and box profile geometries [73,92,100,181] and were incorporated in design standards [111,182]. However, the boundary and interactions between local buckling and compressive failure in terms of the design parameters have not been reported for hollow box PFRP profiles. Studying these interactions can lead to facilitated design guidelines and optimised configurations of the design parameters to fully utilise the profile potentials. In the following sections, these manufacturing design parameters are discussed and the available literature on their effect and interaction is summarised. Moreover, the lack of knowledge and the potential research gaps are highlighted in order to develop the current design for manufacturing manuals.

## 3. Geometric Parameters of Hollow Box PFRP Profiles

The geometric parameters control the PFRP profile stability and determine its load capacity and failure mode [67,183]. These parameters of local buckling are discussed in the following sections by summarising their effect, comparing them for different geometries, and highlighting the available literature on their interactions.

### 3.1. Wall Slenderness

**Figure 8:**
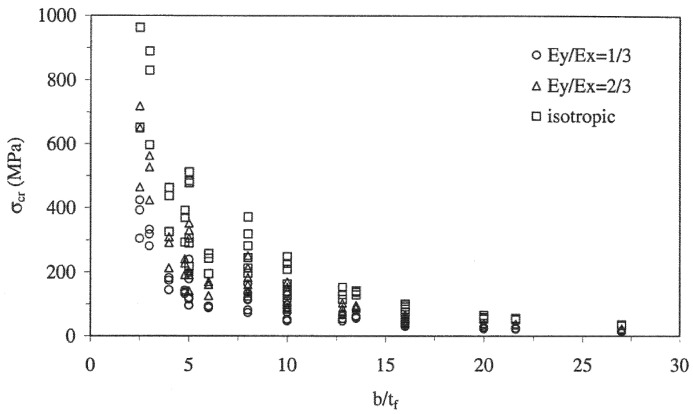
Critical buckling stresses versus the wall slenderness of I-shape PFRP profiles for different levels of orthotropy [81] (Ex and Ey are the longitudinal and transverse modulus, respectively).

The wall slenderness (width-to-thickness ratio) significantly contributes to the local buckling capacity of thin-walled PFRP profiles [77,184]. Reducing the wall slenderness increases the profile stability and buckling capacity exponentially [152,167], and shifts the failure mode from local buckling to material compressive failure due to the increase in the flexural stiffness of the laminated walls [170,185]. The effect of the wall slenderness was studied extensively for laminated plate geometry subjected to uniaxial compressive load [133,143,146,152] and the effect of the layup properties on the buckling load capacity of slender plates was found to be negligible compared to their dimensions [137,144,153]. This finding agrees with the results of parametric studies on open-section PFRP columns [67,81,114], shown in Figure 8. When the slenderness ratio is reduced (thicker walls), the effect of the layup properties becomes significant. On the contrary, the effect of the layup properties becomes negligible when the wall slenderness is increased (thinner walls). Consequently, the layup properties should be considered carefully in the ultimate strength design of thick open-section profiles, while they can be considered only in the serviceability limit (deflection) design of thin open-section profiles [115]. However, the interaction of the wall slenderness with the other geometric parameters and failure modes of box profile geometry was not studied in the available literature.

**Figure 9:**
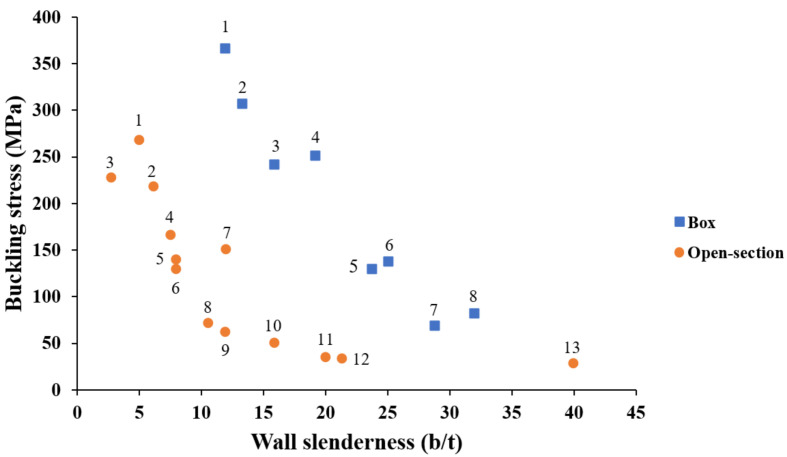
The studied range of wall slenderness for open-section versus box GFRP profiles (Box-shape: 1 [73,92,96], 2 [72], 3 [105], 4 [29,55,56,93,95,103], 5 [85,107], 6 [104], 7 [17], and 8 [75], Open-section: 1 [65,70], 2 [64,72], 3 [75], 4 [67], 5 [62,75,81,83,84,90], 6 [61,72,81,83,84,90,91], 7 [75,77,81,83,84], 8 [61,81,83,84,90], 9 [62,73,83,84,85,90,92], 10 [64,75], 11 [111], 12 [79], and 13 [109,110]).

When comparing the available data, the box profiles exhibited higher buckling capacity compared to the open-section profiles for the same wall slenderness range, as shown in Figure 9. This behaviour can be referred to the higher restraint and torsional rigidity provided on both sides of the wall of box profiles. It was noticed that the thick open-section profiles exhibited a low buckling-to-material strength ratio compared to their counterpart box profiles. Thus, local buckling can be counted as an inevitable failure mode for open-section profiles. On the contrary, local buckling can be avoided for the box profiles if the wall slenderness is slightly increased due to the higher buckling-to-material strength ratio and the available optimisation range. In other words, local buckling can be eliminated in the design for the manufacturing stage, allowing for the ultimate material strength to be used rather than considering the lower buckling strength in the structural design stage of box PFRP profiles. In addition, it was noticed that most of the open-section profiles were widely studied (larger number of references for the same wall slenderness) by experimental, theoretical, and numerical approaches to investigate the wall slenderness. On the contrary, the box profiles had fewer references for the same wall slenderness, which is a sign of few studies assessing the wall slenderness with various methodologies.

Only one study was found to investigate the contribution of multiple design parameters on the local buckling behaviour of pulwound hollow square profiles [94]. The study was conducted on stub columns axially loaded using Taguchi (L9 array) design of experiment, as shown in Table 2 which shows the studied parameters and their levels.

**Figure 10:**
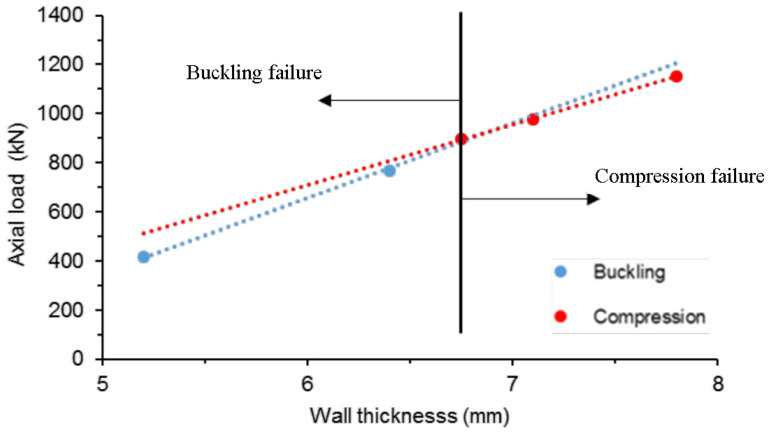
Effect of the wall thickness on the failure mode of pulwound box FRP profile [94].

The resulting compressive strength and stiffness were analysed statistically to rank the effect of these parameters using the signal-to-noise (SNR) ratio and to determine the contribution of each parameter using the analysis of variance (ANOVA). The wall thickness was the dominant parameter for load capacity with a contribution of 93.4%. The winding angle was the second parameter with 2.6% and the axial-to-wound fibre ratio was ranked third with 1.2%. Moreover, the effect of the wall slenderness on the boundary between local buckling and compressive failure of box profiles was reported in this study. The failure mode of the pulwound hollow square profile was estimated to change from local buckling to compressive failure at a wall thickness of 6.75 mm, as shown in Figure 10. However, the interactions between the studied parameters were not captured because of the Taguchi design of experiment limitation (using reduced not full factorial experiment matrix). No study was found to address the relative contributions and interactions of the wall slenderness and the other geometric parameters. Initiating such studies on the design parameters of pulwound box profiles can provide design guidelines and optimal design configurations with improved utilisation, weight, and cost characteristics.

### 3.2. Cross-Sectional Aspect Ratio

The cross-sectional aspect ratio (web height/flange width) defines the unsupported length of each wall and the major and minor axes of the cross-section. It affects the critical buckling load and stability of PFRP profiles [63] and alters their failure mode [186,187,188]. While maintaining a constant cross-sectional area, the flange and web buckling capacities were found to increase and decrease, respectively, when the cross-sectional aspect ratio is increased for both box [63] and open-section beams [172]. 

**Figure 11:**
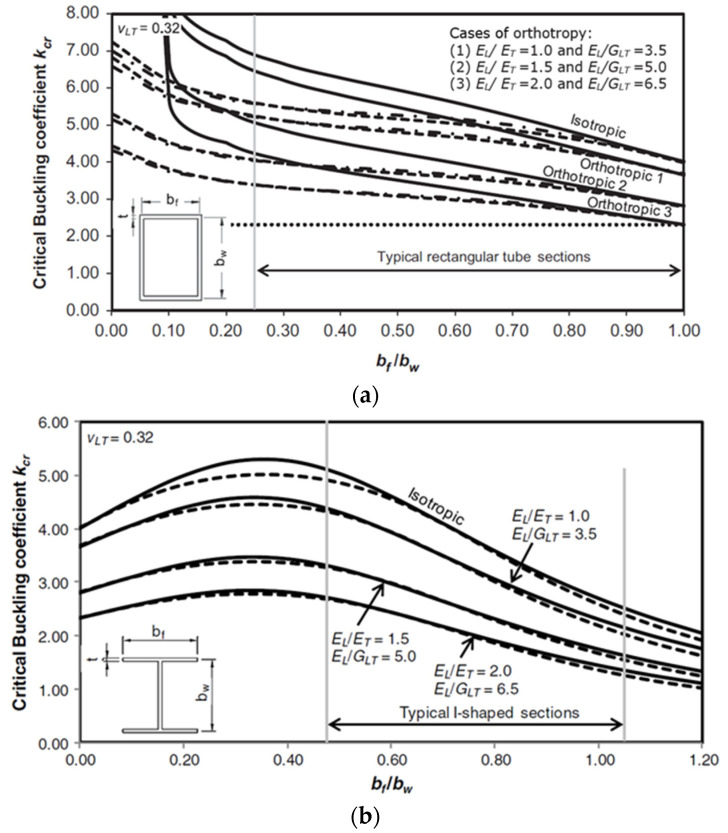
Buckling coefficient (k) versus b_f_/b_w_ for different layup properties of (**a**) box [63] and (**b**) I-shape [64] GFRP columns.

The significant effect of the cross-sectional aspect ratio was characterised under compression and bending for open-section profiles [59,86]. Increasing this ratio three times was found to decrease the buckling strength down to 42.8% under compression while it will increase the buckling strength up to 57.0% under bending. Moreover, the optimal cross-sectional aspect ratios of open-section PFRP profiles were investigated for column [65,109,111] and beam [65,82] applications. In addition, the interaction between the cross-sectional aspect ratio and the layup properties was studied for box [63] and I-shape [64] GFRP columns. The layup properties became insignificant when the flange width was increased and local buckling controlled it, as shown in Figure 11a,b, respectively.

**Figure 12:**
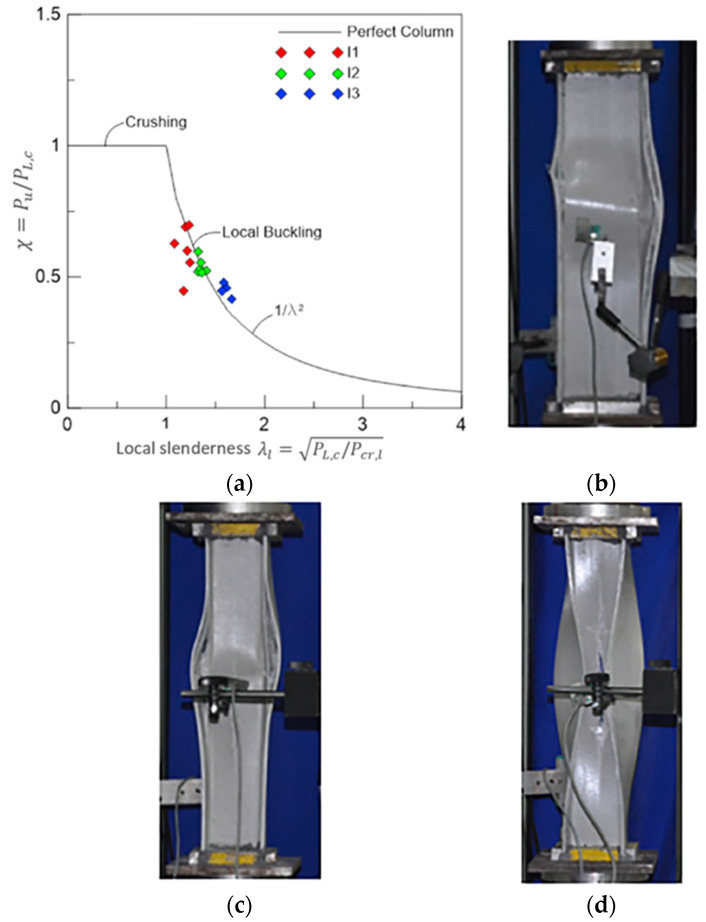
Interaction between local buckling and compressive failure of I-shape PFRP columns with different cross-sectional aspect ratios: (**a**) strength curve with experimental points (P_u_: the ultimate compressive load, P_L,C_: the experimental buckling load, and P_cr,l_: the critical buckling load); (**b**) experimental failure mode of I_1_ (b_f_/d = 0.5); (**c**) experimental failure mode of I_2_ (b_f_/d = 0.75); and (**d**) experimental failure mode of I_3_ (b_f_/d = 1.0) [69].

Moreover, the interaction between compressive failure and local buckling failure modes was studied for box [96] and I-shape [69] GFRP columns. Figure 12 visualises this interaction for I-shape GFRP columns. The first stub column I_1_ (narrow flange) showed an interactive failure mode between compressive crushing of fibres and local buckling of walls (buckling induced material crushing) since it has the lowest local slenderness. On the other hand, the second and third stub columns (I_2_ and I_3_, respectively) failed in local buckling with larger waviness in I_3_ (wide flange). In addition, the boundaries between lateral buckling, web buckling, flange buckling, and interactive buckling failure modes of I-shape PFRP beams were investigated [74]. It was concluded that the interactive (local-lateral) distortional buckling is prominent over the other buckling types and should be considered in the design stage. The interaction of the failure modes influenced the layup properties as the optimal fibre angle was $\theta =\pm 45^{{}^{\circ}}$ against local buckling and was $\theta =60^{{}^{\circ}}-70^{{}^{\circ}}$. against interactive buckling.

**Figure 13:**
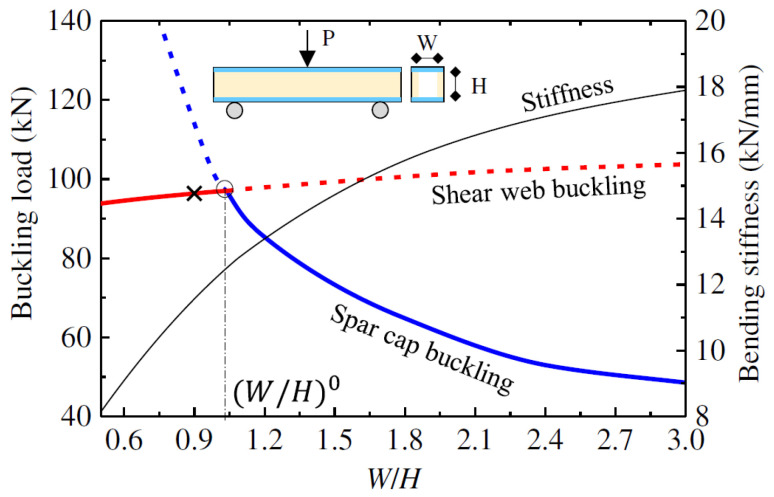
Buckling mode map of GFRP composite box beam with different cross-sectional aspect ratios, where the spar cap is the flange, ×is the baseline design, and O is the desired design [108].

Regarding the box profile geometry, the axial buckling capacity of walls in hollow square beams was reported to be higher than for hollow rectangular beams due to the higher buckling tendency at the weakest direction in the rectangular cross-section [58]. Nevertheless, the overall buckling moment of the beam under bending increases when the cross-sectional aspect ratio is increased since the wall slenderness of the top flange, which carries the majority of the compressive stresses, is decreased [106]. One study was found to examine the interaction between the walls of CFRP box beams [54]. It was reported that webs with a smaller slenderness ratio obtain a higher buckling capacity of the flange due to the higher rotational restraint provided by the thicker webs to the flange. Another study was found investigating the boundary of failure modes of box GFRP beam in terms of the cross-sectional aspect ratio [108]. The effect of the cross-sectional aspect ratio on the buckling of the top flange (spar cap) was significant compared to its effect on the shear web. This was referred to the higher compressive stresses acting on the top flange, which made its buckling load more sensitive to the change of dimensions. The optimal buckling capacity was obtained at the inflection point of the flange buckling and web buckling failure modes, which is denoted by the “○” symbol in Figure 13. This point represents the best cross-sectional aspect ratio for maximum buckling capacity and minimum material usage of the beam.

**Figure 14:**
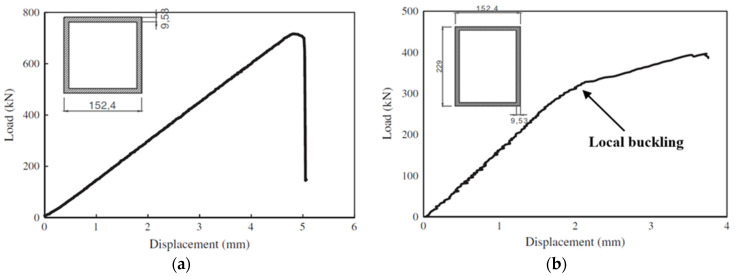
Load-displacement curves of hollow (**a**) square and (**b**) rectangular box PFRP profiles under axial compression [17].

Rectangular box profiles (with web height/flange width ≥ 1.5) were found to exhibit a post-buckling trend in their load-displacement curves under compression loading [17]. Figure 14 compares the load-displacement curves of hollow square and rectangular PFRP profiles subjected to axial compression. The hollow square profile exhibited linear elastic behaviour until the peak (buckling) point, then failed. On the other hand, the hollow rectangular profile showed a linear elastic behaviour until the buckling point of the wider walls then the structural stiffness was degraded due to the loss of stability of the wider walls and the load capacity increased under a new equilibrium path until failure occurred. Although the cross-sectional area of the rectangular profile is 26.9% higher than for the square profile, its buckling strength was 54.7% less than the square profile due to the higher wall slenderness of the wide walls, which caused earlier buckling and suppressed the profile potentials. However, no study was found to address the interactions between the cross-sectional aspect ratio and the other geometric parameters, or the effect of the interaction between the flange and webs on the stability and overall structural behaviour of pulwound box PFRP profiles. Such studies can provide optimal design configurations and better design guidelines as the current design formulas are conservative and consider only the wall with the maximum slenderness ratio for buckling capacity estimation and do not include the interaction between the flange and the webs and their corner radius.

### 3.3. Corner Geometry

**Figure 15:**
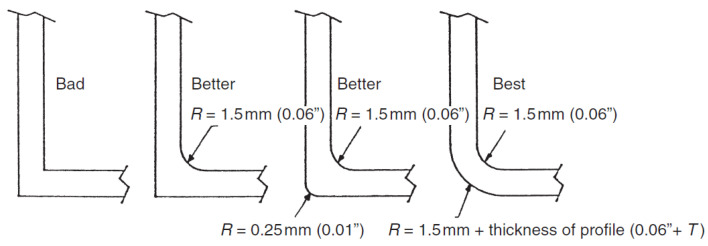
Recommended configurations of the corner of PFRP profiles [192].

The corner (flange-web junction) geometry of PFRP profiles is a critical manufacturing parameter affecting the production process, the pulling force, and the heated die settings. It is considered to be a weak point of premature failure due to stresses concentration at this critical zone [189,190,191]. It is recommended to increase the inner corner radius (fillet) to prevent cracking by uniformly distributing the stresses and preventing their concentration [192], as shown in Figure 15. Increasing the outer corner radius to be equal to the inner radius plus the wall thickness can also facilitate the production process and help to avoid thermal-induced cracks [192]. 

**Figure 16:**
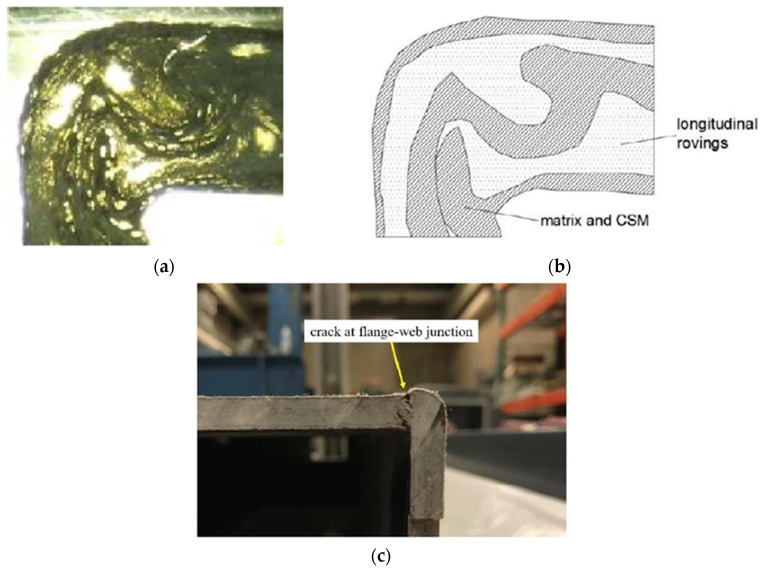
Flange-web junction of hollow box pultruded GFRP beam (102 mm × 152 mm × 6.4 mm): (**a**) image of fibre and matrix architecture, (**b**) schematic of fibre and matrix architecture, and (**c**) crack at the flange-web junction under bending [101].

One study was found to experimentally characterise the structural behaviour of the corner of commercial box GFRP beams with longitudinal glass rovings and continuous strand mat (CSM) layups [101]. Microscopic photos were taken to diagnose any resin-rich zones and fibre wrinkling, as shown in Figure 16a,b. Although these manufacturing defects were distributed along the walls, the failure of box GFRP beams initiated at the corners due to the discontinuity in fibres and stresses concentration was noticed, as shown in Figure 16c. It was recommended that the steep change in the inner corner geometry could be changed from right angle to fillet in order to uniformly distribute the stress between the walls.

**Figure 17:**
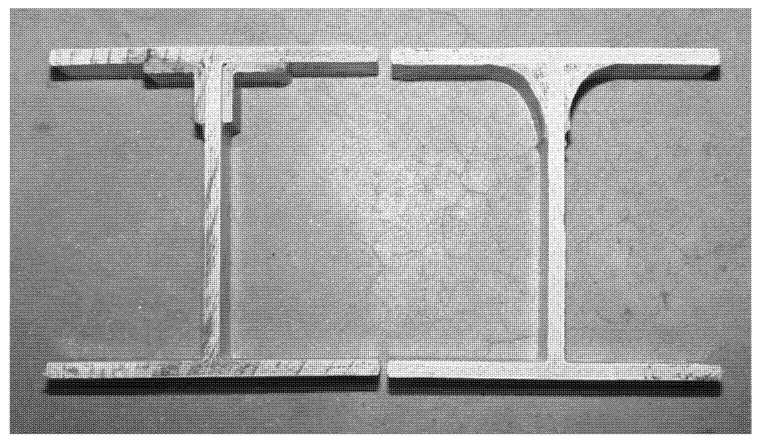
Corners of I-shape PFRP beam (203 mm × 203 mm × 9.5 mm, radius 2.38 mm) enhanced by (left side) polyester pultruded equal leg angles (38 mm × 38 mm × 6.4 mm) and (right side) hand-layup fillets (38 mm) on the top corners [61].

Regarding the local buckling behaviour, the corners (initial radius 2.38 mm) of open-section (I-shape) PFRP beams were enhanced by bonding polyester pultruded equal leg angles (38 mm × 38 mm × 6.4 mm) or hand-layup fillets (38 mm) on the top corner [61], as shown in Figure 17. In both cases, the load capacity was significantly enhanced by 1.5 times due to the increased geometry, which enhanced the rotational stiffness and strength of the corners and allowed for uniform distribution of stresses. The failure mode was shifted from buckling of the top flange to compressive failure of fibres with the ultimate material strength fully utilised. In another study, CFRP layers and GFRP stiffening plates were used to strengthen the corners of I-shape beams to increase their buckling capacity [89]. This approach was proven to be very effective in preventing local buckling of the flange and enhancing the flange-web junction and the flexural strength of the beams. In these two studies, the fillet geometry exhibited a better effect than angles and plates due to the lower stresses concentration caused by their uniform change of geometry compared to the sudden change in the cross-section of the beam caused by the angles and plates.

However, no study was found to address the inner and outer corner radii effect as manufacturing parameters on the local buckling capacity of PFRP profiles. In addition, no study was found to address the corner geometry effect on local buckling of box PFRP profiles. Moreover, the effect and interaction of the layup properties on the corner radii have not been studied for box profiles since most of the reported investigations on the layup parameters considered laminated plate geometry. In addition, the effect of continuous confinement provided by the wound fibres around the corners in pulwound box profiles has not been reported. Currently, standards and design manuals do not include the corner radius as a design parameter in their equations and structural designs. Moreover, the corner geometry (e.g., inner-to-outer radii ratio) needs to be investigated to reflect its contribution to the local buckling capacity in the related design equations. Consequently, understanding the corner geometry role as a design parameter for local buckling will lead to more stable designs of box PFRP profiles with enhanced load capacity and the avoidance of buckling failure.

## 4. Layup Parameters of Hollow Box PFRP Profiles

The layup properties define the anisotropy and mechanical properties of FRP profiles in the longitudinal and transverse directions and directly affect their local buckling behaviour [193]. These properties should be designed depending on the intended application since the design will address a specific geometry and loading condition and cannot be generalised for all composite structures [143,194]. The layup parameters of local buckling are discussed in the following sections by summarising their effects, comparing them for different geometries, and highlighting the available literature on their interactions.

### 4.1. Axial-to-Inclined Fibre Ratio

For civil structural applications, the layup of PFRP profiles consists of longitudinal fibre rovings to obtain the required axial and flexural stiffness and off-axis (inclined) fibres to enhance the shear and transverse properties [42,195]. The ratio of these axial-to-inclined fibres shapes the anisotropy and mechanical properties of the laminated walls to achieve the required axial and flexural stiffness and the desired shear and transverse properties. In general, it is recommended to add inclined fibres along with the axial plies to enhance the off-axis mechanical properties, damage tolerance, and stability of laminated plates [196,197]. These inclined fibres are also needed to fulfil the web stiffness and strength requirements of PFRP beams [198,199]. 

Regarding the geometry effect on this ratio, it was found that increasing the axial fibre percentage will increase axial buckling resistance of laminated plates [123]. On the contrary, increasing the inclined fibre percentage will increase the local buckling strength of open-section FRP columns due to the higher rotational rigidity between the orthogonal walls [200]. No study was found on the interaction between the axial-to-inclined fibre ratio and the other layup properties or on its effect on the geometric parameters of pulwound box FRP profiles.

### 4.2. Inclined Fibre Angle

In classical laminated plate theory (CLPT), FRP composite plates with angle-ply (${\left[\pm \theta \right]}_{S}$) layup exhibit the maximum local buckling capacity at a fibre angle (θ) of $\pm \;45^{{}^{\circ}}$ since it obtains the highest bending-extension stiffness parameters (Dij) [153,201]. However, axial fibre rovings must be added to meet the axial and flexural stiffness requirements for civil structural applications. Moreover, it was proven that introducing new fibre angles apart from the traditional $0^{{}^{\circ}},\;\pm 45^{{}^{\circ}}$, and $90^{{}^{\circ}}$ angles can also provide improved designs for local buckling of different geometries and loading conditions [147]. The contribution of the fibre angle on the buckling capacity was found to be significant for certain geometries. For instance, small fibre misalignments, such as $\pm 2^{{}^{\circ}}$, were noticed to affect the buckling capacity of GFRP tubes up to 7.8% [161]. 

The optimal fibre angle to obtain the maximum buckling capacity is a function of the geometry, boundary condition, and loading condition [133,143,194]. Under flexural loading, it was found that increasing the web orthotropy exhibits the highest increase in the buckling capacity of the flange due to the increase in the rotational restraint at the flange-web junction. Moreover, the increase in the flange buckling capacity is higher when its orthotropy is low [54]. For open-section FRP beams, the buckling load was found to decrease when the fibre angle is increased [58]. 

**Figure 18:**
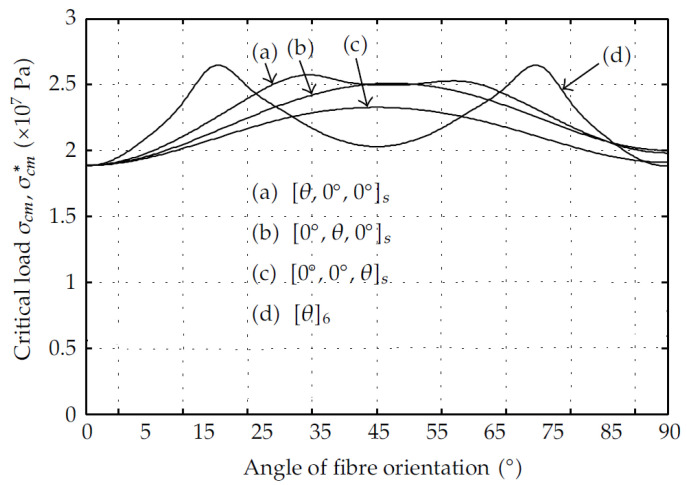
Critical buckling load versus varying inclined fibre orientation for different stacking sequences in GFRP cylindrical shells [167].

Moreover, the interaction between the fibre angle and the stacking sequence was found to be significant and may shift the optimal fibre angle depending on the geometry and boundary and loading conditions [149,168]. For instance, antisymmetric laminated plates require a fibre angle of $25^{o}$ to obtain the maximum buckling load unlike symmetric laminates [157]. Even for symmetric layups, the optimal fibre angle for maximum buckling of GFRP cylindrical shells changes depending on the introduction or removal of axial fibres [167], as shown in Figure 18. Stacking the inclined plies at the outer side to confine the axial fibres enhances the buckling capacity. Regarding the pulwound FRP profiles, no study was found to investigate the winding angle effect on the corner geometry or its interactions with the other layup parameters under compression or bending. Assessing the contribution of this parameter on the buckling resistance of pulwound box PFRP profiles will alleviate the lack of knowledge for this special shape.

### 4.3. Stacking Sequence

**Figure 19:**
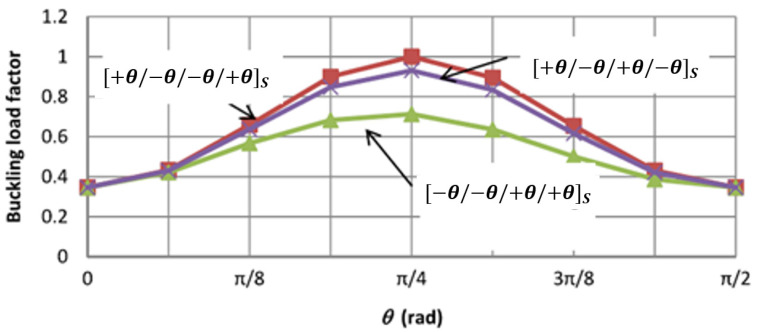
Effect of fibre angle on the buckling load of simply supported laminated plate with symmetric stacking sequences [209].

The stacking sequence of laminated composites affects their stability, deflection response, interlaminar stresses, post-buckling behaviour, and progressive failure [202,203,204]. Its optimal configuration to resist local buckling depends on the geometry and boundary and loading conditions and has to be determined specifically for the intended application [138,205]. In general, stacking the inclined plies to the outer surface of a laminated plate enhances the local buckling resistance under axial compression due to the increase in confinement [162,206]. On the contrary, stacking the axial fibres to the outer surface increases the plate buckling resistance against transverse compression [207]. A compromise between the buckling capacity and other mechanical properties should be considered in the design since stacking axial fibres at the outer surface exhibits higher tensile and flexural moduli [208]. In general, stacking sequences with elastic coupling are not preferred for compressively loaded members as they are vulnerable to manufacturing imperfections, buckling, bending, and warping due to thermal effects [120,196,207]. Thus, symmetric and balanced layups are usually used to minimise the coupling effects. For simply supported laminated plates, the interaction between the stacking sequence and fibre angle was found to be significant at $\theta =45^{{}^{\circ}}$ [209], as shown in Figure 19. The minimum buckling load was obtained when the −θ plies were outmost from the mid-plane due to the maximum effect of bending-twisting coupling (maximum value of $D_{16\;}+D_{26\;}$). The reduction in the buckling load for this case reached its peak at $\theta =45^{{}^{\circ}}$ with a 25% drop in load from the optimal case (${\left[+\theta /-\theta /-\theta /+\theta \right]}_{S}$).

**Figure 20:**
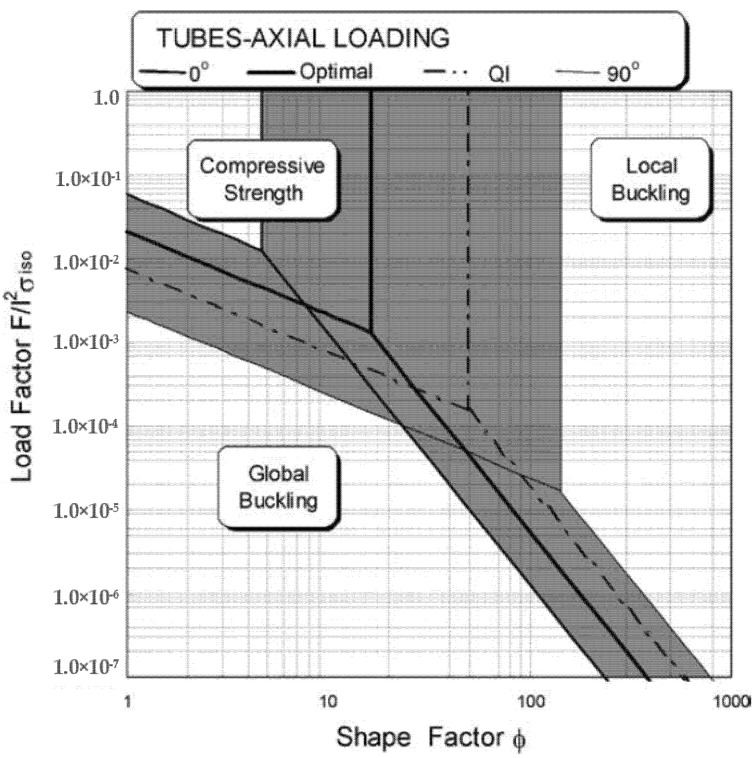
Failure chart of CFRP composite cylindrical shells as a function of the shape factor (wall slenderness = radius/thickness) and stacking sequence ($0^{{}^{\circ}}$: axial fibre layup, $90^{{}^{\circ}}$: transverse fibre layup, QI: quasi-isotropic layup, and optimal: ${\left[55/-55/0_{6}\right]}_{S}$) [160].

Regarding the geometry effect, the stacking sequence was found to affect the boundaries of different failure modes of CFRP composite cylindrical shells [160], as shown in Figure 20. Reducing the shape factor (radius/thickness) shifts the failure mode from local buckling towards compressive failure. The $0^{o}$ laminate possesses the maximum axial compressive strength and the largest local buckling failure zone because of the axial direction of the fibres and the minimum circumferential confinement (maximum out-of-plane waviness), respectively. Conversely, the $90^{o}$ laminate exhibits the minimum axial compressive strength and the smallest local buckling failure zone because of the transverse direction of the fibres and the maximum circumferential confinement (minimum out-of-plane waviness), respectively. The ${\left[55/-55/0_{6}\right]}_{S}$ laminate presents the optimal compromise against both local and global buckling. On the contrary, angle-ply laminates with $\pm \;25^{o}$ and $\pm \;90^{o}$ plies possess the highest local buckling strength for CFRP cylindrical shells with geometric imperfections [159]. When comparing cross-ply and angle-ply layups for laminated plates under uniaxial compression, cross-ply layups exhibited optimal buckling resistance [138,183] while for cylindrical shells angle-ply is better [155]. For open-section profiles, angle-ply laminates obtained a higher buckling load than quasi-isotropic laminates [113]. The buckling capacity of these profiles was decreasing when the fibre angle was increased and the cross-ply laminates were observed to sustain a larger buckling load than angle-ply when the fibre angle is larger than $30^{{}^{\circ}}$ [58,80]. It was found that the effect of the stacking sequence on the buckling capacity of laminated plates decreases as their dimensions are increased [128] but it becomes significant in open-section structural-level columns with slender walls [140]. No study was found on the effect of stacking continuous wound fibres with different sequences on the corner geometry of pulwound box PFRP profiles, or on the interaction between the stacking sequence and other layup parameters in such profiles.

## 5. Conclusions

Hollow box PFRP profiles are increasingly used as structural elements in civil structural applications. Although the studies and the standards were developed to facilitate the design process of PFRP profiles, there is still a lack of knowledge regarding the local buckling design parameters (layup and geometry) for box profile geometry. This presents an issue in designing these profiles and fully using their potentials, evident by the limited range of specifications in the available commercial profiles. This article presents a literature review on the local buckling design parameters controlling the structural behaviour of box PFRP profiles. Although most of these parameters were studied individually, there is still a need to perform a comprehensive study to obtain their contribution and interaction, which will provide practical design guidelines and recommended configurations of the design parameters. This review on the design parameters of PFRP profiles outlines the current state of knowledge and the investigations to be conducted. Thus, it provides a useful reference to researchers and design engineers. Furthermore, it presents a benchmark for the next generation of design guidelines, which will broaden the use of PFRP in construction by eliminating the current difficulties in PFRP profiles design. Based on this review, the current state of knowledge and future trends for optimising these profiles and their design parameters are summarised as follows:

- Hollow box PFRP profiles are featured with higher structural stability and torsional rigidity compared to the open-section profiles due to the restraint at both ends of the wall and its unique stresses distribution. However, their design parameters have not been studied comprehensively as for open-section and laminated plate geometries. While local buckling is inevitable for open-section profiles, it can be avoided for box profiles if the wall slenderness is optimised due to the high buckling-to-material strength ratio and the available optimisation range. This will allow the design to consider the ultimate material strength rather than considering the lower buckling strength.
- The flange-web junction (corner) radius and its effect on the local buckling of hollow box PFRP profiles have not been studied or quantified even though its effect was significant on the buckling behaviour and failure mode of open-section profiles. Moreover, the interaction between the layup properties or the flange-web slenderness and the corner geometry has not been studied for box profile geometry. In addition, the effect of continuous confinement provided by the wound fibres around the corners in pulwound box profiles has not been reported. The corner (fillet) radius is not included in the analysis and design equations of box PFRP profiles. No study was found to address the inner and outer corner radii effect on the local buckling capacity as manufacturing parameters of PFRP profiles.
- Pulwound box FRP profiles were recently introduced for infrastructure applications with better transverse and circumferential properties. However, studies are still needed to comprehensively address all the critical design parameters controlling the local buckling of these profiles and quantify their relative contributions and interactions. Considering these interactions can facilitate economic structural designs and guidelines for these profiles, eliminate any conservative assumptions, and update the current design standards and manuals. Understanding the contributions and interactions of these parameters will broaden the use of these profiles with competitive structural performance and cost versus the conventional construction materials.
- As with the other structural shapes, there is a need to construct design curves and failure maps for hollow box PFRP profiles, considering the interactions and showing the shift in the failure modes in terms of the critical design parameters. Investigating these review findings, especially the importance of the interactions, will enhance the current design guidelines, facilitate economic and competitive designs, and manufacture optimised profiles for civil structural applications.

## Figures and Tables

**Table 1:** Local buckling design formulas of compression box and I-shape members in current standards and guides.

Design Standard	Considered Geometry	Design Formula ^1^
Pre-standard for load & resistance factor design (LRFD) of pultruded fibre-reinforced polymer (FRP) structures [177]	Hollow box	$\left(f\right)=\frac{\left(\frac{\pi^{2}}{6}\right)\left[\sqrt{E_{L}E_{T}}+v_{LT}E_{T}+2G_{LT}\right]}{{\left(\frac{b}{t}\right)}^{2}}$
	I-shape	${\left(f\right)}_{flange}=\frac{G_{LT}}{{\left(\frac{b_{f}}{2t_{f}}\right)}^{2}}$
Prospect for new guidance in the design of FRP [178]	Hollow box	$\left(f\right)=\frac{\pi^{2}}{b^{2}t}\left[2\sqrt{\left(1+4.139\zeta \right)\left(D_{11}D_{22}\right)}+\left(2+0.62\zeta^{2}\right)\left(D_{12}+2D_{66}\right)\right]$ Where: $\zeta ={\left(1+\frac{5}{1-R}\frac{{\left(D_{22}\right)}_{f}b_{w}}{{\left(D_{22}\right)}_{w}b_{f}}\right)}^{-1}$$,\;R=\frac{{\left(f\right)}_{f}{}^{ss}{\left(E_{L}\right)}_{w}}{{\left(f\right)}_{w}{}^{ss}{\left(E_{L}\right)}_{f}}$
	I-shape	Same as [180]
Structural Design of Polymer Composites EUROCOMP Design Code and Handbook [179]	Orthotropic plate	$\left(f\right)=2\pi^{2}\frac{\left(\sqrt{D_{11}D_{22}}+H_{o}\right)}{tb^{2}}$ Where: $H_{o}=0.5\left(v_{LT}D_{22}+v_{TL}D_{11}\right)+\frac{G_{LT}t^{3}}{6}$
Guide for the Design and Construction of Structures made of FRP Pultruded Elements [180]	I-shape	${\left(f\right)}_{flange}=\{\begin{array}{c} \frac{\sqrt{D_{11}D_{22}}}{t_{f}{\left(\frac{b_{f}}{2}\right)}^{2}}\left(K\left[15.1\eta \sqrt{1-\rho }+6\left(1-\rho \right)\left(1-\eta \right)\right]+\frac{7\left(1-K\right)}{\sqrt{1+4.12\zeta }}\right),\;K\leq 1\\ \frac{\sqrt{D_{11}D_{22}}}{t_{f}{\left(\frac{b_{f}}{2}\right)}^{2}}\left[15.1\eta \sqrt{1-\rho }+6\left(1-\rho \right)\left(K-\eta \right)\right],\;K>1 \end{array}$ Where: $\zeta =\frac{D_{22}}{\tilde{k}\frac{b_{f}}{2}}$$,\;\rho =\frac{D_{12}}{2D_{66}+D_{12}}$$,\;\eta =\frac{1}{\sqrt{1+\left(7.22-3.55\rho \right)\zeta }}$$,\;K=\frac{2D_{66}+D_{12}}{\sqrt{D_{11}+D_{22}}}$

^1^$\;D_{11}$, $D_{22}$, $D_{12}$, and $D_{66}$ are the flexural rigidities of the orthotropic plate. $\tilde{k}$ is the torsional stiffness coefficient. *t*, *b,* and *h* are the section thickness, width, and height, respectively. The subscripts *f* and *w* refer to the flange and web, respectively. $E_{L}$, $E_{T}$, and $G_{LT}\;$ are the longitudinal, transverse, and in-plane shear elastic moduli, respectively. $v_{LT}$ and $v_{TL}$ are the in-plane and out-of-plane Poisson’s ratios, respectively. ${\left(f\right)}_{f}{}^{ss}$ and ${\left(f\right)}_{w}{}^{ss}$ are the buckling strengths of the flange and web, respectively, considering simply supported boundary conditions.

**Table 2:** Parameters and levels investigated in Alsaadi 2019 [94] parametric study.

Profile Dimensions	Parameters	Level 1	Level 2	Level 3
Section (mm): 100 × 100	Wall thickness (mm)	5.2	6.4	7.8
Corner radius (mm): inner 4.8 and outer 10	Winding angle (degrees)	45	60	75
Height (mm): 500	Axial-to-wound fibre ratio (%)	80/20	70/30	60/40

## Data Availability

The data presented in this study are available on request from the corresponding author.
